# Expanding the role of paralegals: supporting realization of the right to health for vulnerable communities

**DOI:** 10.1186/s12914-020-00226-y

**Published:** 2020-03-30

**Authors:** Albert Wirya, Ajeng Larasati, Sofia Gruskin, Laura Ferguson

**Affiliations:** 1 Lembaga Bantuan Hukum Masyarakat (Community Legal Aid Institute), Tebet Timur Dalam VI E No 3, Jakarta, 12820 Indonesia; 2 USC Institute on Inequalities in Global Health, University of Southern California, 2001 N Soto St, Los Angeles, USA

**Keywords:** Paralegal, Access to justice, Right to health, Vulnerable communities, Legal empowerment

## Abstract

**Background:**

All around the world, the paralegal program prepares members of marginalized communities to face the legal system. Having a common background with their clients and being capable of conducting flexible work, paralegals’ role moves beyond enlarging the beneficiaries of legal aid to addressing intersectional issues around health-related rights. This study assesses the health and other impacts of paralegals recruited by Lembaga Bantuan Hukum Masyarakat (LBHM), a human rights organization which provides legal assistance and operates in Jakarta, Indonesia. The positive results these paralegals can bring in the specific context can contribute to the development of community-based paralegals elsewhere.

**Methods:**

This mixed methods research was carried out in 2016–2018. In 2016, a quantitative survey was administered to LBHM-trained paralegals and a sub-set of paralegals who completed the survey were then also interviewed. Quantitative data were analysed using SPSS, and, for the qualitative data, thematic analysis was conducted.

**Results:**

The paralegals make important contributions to health-related rights in four distinct ways. Firstly, most of the paralegals checked their clients’ health in all stages of detention, especially regarding their drug dependency status and checking for signs of torture. Secondly, paralegals help clients to be more aware of their health-related rights, especially regarding their rights to obtain health services inside detention. Thirdly, paralegals can ensure that their clients obtain health services by taking medicines directly to the clients or encouraging the law enforcement agencies to refer the clients to health services. Lastly, in drug cases, paralegals help their clients to obtain alternative sentences besides imprisonment.

**Conclusions:**

These four contributions verify the positive impacts paralegals, recruited from marginalized communities, can deliver for community members facing criminal justice processes. The shifting role of paralegals from merely an intermediary between clients and lawyers to champions of the health-related rights of their clients can happen as a result of adequate training, support, and networks with other agents in criminal justice system.

## Background

Paralegal programs often fall within the rubrics of access to justice and legal empowerment. Access to justice approaches generally attempt to expand access to just treatment under law while legal empowerment has a specific focus on empowering poor, vulnerable and/or excluded populations, and increasing the capacity of communities to exercise their own rights [1, 2]. Appropriately trained community-based paralegals can: provide invaluable assistance for legal education [3], navigate the legal systems [4], and provide support throughout the legal process [5, 6].

Traditionally a paralegal has been defined as “A person who has been trained, and holds authority to provide a specified number of legal services. A paralegal is not a lawyer, but is usually on their way to becoming one” [7]. The NGOs which have paralegal programs selected their paralegals using several common criteria, including being trusted by the community, being involved in community-based activities and having experience in advocacy or legal aid. In most projects, they are trained to assist clients in accessing legal institutions including lawyers and the judiciary by acting as a liaison [8]. In 2007, it was found that the majority of work undertaken by paralegals in Indonesia was for the poor and connected to legal assistance (40%), followed by advocacy (29%) and finally mediation (19%) [9].

However, the role of paralegals is expanding because of their flexible work and affiliations. Moving beyond targeting those who face economic [10] and physical [11, 12] barriers for obtaining legal aid, paralegals around the world also address intersectional issues around health-related human rights. Several programs exist through which paralegals assist people who use drugs [6], sex workers [13], and people living with HIV [6]. As these groups of clients have specific health concerns, paralegals’ work might not be limited to only access to justice or legal empowerment, but also to the advancement of the right to health and other health-related rights. Health-related rights as used here refers not only to the right to health but other rights that intersect with the fulfilment of the right to health, such as the right to not be tortured, non-discrimination, the right to information, etc.

Given the inter-related nature of these rights, by providing access to justice and legal empowerment, paralegals can simultaneously enhance health-related rights. Other academic work regarding paralegal interventions in Asia, Africa and Europe have initiated the discussion about the health benefits of having paralegals involved in legal cases. For examples significant health benefits have been found from paralegals’ work to prevent torture in detention in Ukraine [13] and advocating for pre-trial release of their clients in Malawi [5]. Paralegals can also give additional support specifically to the benefit of their clients’ health during the legal process. Paralegals have become the providers of food or clean needles in detention facilities [13]; and, for example, in Kyrgyztan they persuaded law enforcement agencies to understand and address detainees’ health problems [6].

Paralegals can also have impacts outside the criminal justice system that support the health of their clients. Among marginalized Roma communities, paralegals have helped clients to face the refusal of care, extortion, and other abuses from health care workers [14]. In South Africa, paralegals have assisted their clients in accessing government programs that are associated with health care access, such as disability funds and other social safety nets [15]. This breadth of work shows that paralegals are not merely replacements for lawyers; in many cases, they move beyond what lawyers can do in providing more assistance related to health.

While the impact of paralegals on health-related rights seems promising, the evidence is less well documented. In this study, we seek to better understand the impact of paralegals’ work on the health-related rights of key and vulnerable populations including people who use drugs by considering a specific well-documented program in Jakarta, Indonesia. With implications for other similarly situated populations, particularly in resource limited settings with repressive legal environments, this project constitutes a case study of how members of a community living in conflict with the law can be trained as paralegals to improve access to justice and how they also had a positive impact on health-related outcomes. This Indonesian case study can contribute to a global and ongoing conversation about how paralegal interventions can shape the concept of the legal aid, not merely as a legal representative but also with respect to the fulfilment of inter-related rights during the criminal justice process.

### The Indonesian context

In Indonesia, detention is a common norm for suspected criminals. Although bail is possible, law enforcement agencies seldom grant it. During standard criminal justice processes, detention usually takes place in three different settings. The first is police detention which is managed by the police. The second is detention centers managed by the Prison Department for suspects who are being prosecuted by the district attorney or awaiting trial. Both of these detentions combined can result in up to two hundred days of pre-sentencing detention. The third detention setting is the correctional institution, the duration of which is determined by the judge’s verdict.

Health risks in detention are elevated in the Indonesian context where most prison and detention centers are overcrowded, ranging from 80 to 300% over maximum capacity [16]. One reason for overcrowding is that many defendants are unable to obtain bail, hence serving pre-trial detention up to 200 days before sentencing. In addition to that, more than half of prisoners are sentenced for drug offences, which carries a minimum 4 years sentence, where many of them have to serve full time sentence due to limited access to parole. In the context of this lengthy detention, the role of paralegals becomes particularly crucial. This role then includes ensuring that health facilities and services are accessible to their clients without discrimination.

### The LBHM paralegal program

Lembaga Bantuan Hukum Masyarakat (LBHM) is a non-governmental organization which advocates for human rights in Indonesia. LBHM was established with a belief that every single person can be an advocate for themselves if they are equipped with proper knowledge and skills. Based in the capital, Jakarta, this organization gives law and human rights training to marginalized communities, such as people who use drugs, people living with HIV (PLHIV), LGBTQI, sex workers, and other communities. Prior to the training, LBHM deploys a team of legal counsellors to assess the situation of the target communities, as well as to identify common legal and human rights issues that the community faces. This process can involve up to three meetings.

After the assessment, the legal counsellor team will draft a simple syllabus for law and human rights training specific to the community’s needs. Based on the experiences of many paralegals who themselves have faced stigma and discrimination impacting their health and in accessing health services, the principles of the right to health, and interaction with other health-related rights frequently became a necessary part of the training, alongside other compulsory material, such as the basic of human rights, the right to a fair trial, and criminal proceedings. Tailored to the community, LBHM might add some sessions e.g. on drug laws, or understanding bylaws that criminalize sex work.

During this series of trainings, LBHM tries to identify potential community members to be further trained as paralegals. Since 2008, LBHM has recruited and trained around 81 paralegals. Following a traditional understanding of the role of paralegals, the initial idea was that the paralegals would bridge members of the most marginalized and vulnerable communities to legal service providers, such as legal aid organisations or pro bono lawyers for further legal advice or assistance. Paralegals can provide early legal interventions in the form of basic advice on the “dos and don’ts” following arrest, prior to bringing the case to legal aid organisations or pro bono lawyers for a more in-depth intervention.

Although originally designed to address legal issues faced by communities, the work of paralegals often benefits the clients’ state of health through contributions to realizing their health-related rights. Most of the paralegals recruited by LBHM come from communities with specific concerns around health that often stem from insufficient attention to their health-related rights, such as people who use drugs and sex workers. When people from these communities face criminal charges, their health-related rights, such as their right to not to be tortured or their right to information, are often unfulfilled. Using their expertise gained in the community, paralegals can advocate for the realization of health-related rights, including through improving the availability and accessibility of good-quality health services.

## Methods

### Aim

The aim of this study was to assess the impact of LBHM’s paralegals’ work, including with respect to health-related rights, and by extension the health of the people they serve, in the community, in closed-settings and during the criminal justice process.

### Data collection and analysis

Data were collected between August and December 2016. All participants are community-based paralegals recruited by LBHM. All paralegals in the database were contacted and asked to participate in the research.

Data were gathered in two ways. The researchers administered a quantitative questionnaire, developed specifically for the purpose of this study, to measure the general outcomes of their work, especially regarding the right to health, and their methods of working. The questionnaire covered: the types of services paralegals provided during case assistance, especially during litigation case advocacy as well as the activities paralegals carried out outside the criminal justice process. In the following 2 months after the collection, Statistical Package for Statistical Package for Social Science (SPSS) was used to calculate frequencies for different components of the paralegals’ work.

Initial quantitative findings were used to inform an interview guide for qualitative data collection to explore paralegals’ experiences in greater depth with a view to providing more detailed insight into how the paralegals have helped their communities. During March to May 2017, semi-structured interviews were carried out with six paralegals drawn from respondents to the quantitative survey. The researcher, a local researcher working with LBHM, explored the specific cases paralegals had handled and the impacts of this work focusing on the information about the paralegals’ impact on clients’ access to justice and to health services. Qualitative data were thematically analysed.

Written informed consent was provided by all of the research participants following the provision of information about the study. For confidentiality reasons, all qualitative interview participants were assigned a number, which will be used to refer to them throughout this article. Pseudonyms are used for all people referred to in direct quotes from the paralegals.

## Results

### Participants

Questionnaires were successfully administered to 24 paralegals. The number is relatively low from the estimated number of all paralegals LBHM has trained (81 people) because many of them have moved away or could not be contacted. There are many potential reasons why it was difficult to contact some paralegals, including simply that the time between when the training was offered and when the research was conducted was quite long, as well as potentially ineffective coordination.

An overview of the characteristics of the paralegals who participated in this research is provided in Table 1.

**Table 1:** Characteristics of the study population

Variable	Persons (*N* = 24)
Gender	
Male	22
Female	2
Age	36 (22–75)
Occupation	
Employee	3
Fisherman	4
Hospital Peer Support Staff	1
Local CHC Staff	2
Nongovernmental Organization Staff	2
Self-employed	2
Student	2
Unemployed	3
Other informal sectors	5
Year of Paralegal Training	
2008	18
2011	1
2013	2
2015	2
2010	1

Not all paralegals have had experience in working on both access to justice and access to health. Some cases that are handled by paralegals do not involve health issues or problems. Based on themes that emerged from the research, findings are divided into four categories below: checking the clients’ health, raising clients’ awareness regarding the right to health, ensuring health services in detention, and seeking an alternative to imprisonment or lower sentence in legal defense.

### Checking the clients’ health

Under Indonesian law, police investigators, prosecutors, and judges can place an accused in detention if the sentence for the crimes is more than 5 years, or if there is any indication that the accused will run away, destroy evidence, or commit other crimes. Therefore, detention is the most common setting where paralegals meet with their clients. It is during the meeting in detention that paralegals check their clients’ health condition. Not all of the paralegals interviewed had experience assisting clients who were detained. Thirteen paralegals had experience in assisting clients who were in police detention, while only eight had experience in assisting clients who were held in a detention center or a correctional facility. The remaining paralegals had either never met a client in person, or had handled private legal cases.

The client’s health condition was one of the first pieces of information that the paralegals sought during assessment. Sixty nine percent^1^ of paralegals who had assisted clients in police custody reported having checked on a client’s health condition in police custody though not all of them did so consistently. Meanwhile, 87.5% of paralegals who had assisted clients in detention centers and correctional facilities checked on the health condition of clients who were detained in these particular settings. Further explanations can be viewed on the Table 2.

**Table 2:** Paralegals check on the health condition

Settings	Responses					Total
	Never	Seldom	Sometimes	Frequently	Always	
Police Detention	4 (31%)	1 (8%)	0 (0%)	3 (23%)	5 (38%)	13 (100%)
Detention Center	1 (12.5%)	1 (12.5%)	1 (12.5%)	2 (25%)	3 (37.5%)	8 (100%)
Correctional Facility	1 (13%)	1 (13%)	1 (13%)	3 (39%)	2 (26%)	8 (100%)

When asked what situations led to them checking on a client’s health condition, paralegals who did not always ask about the client’s health condition said that it usually depends on the case assisted and the nature of the client. For example, if paralegals know that the client is a drug user, or if there are physical signs or information that the client has experienced torture, they will ask about the health condition.

Yes, if I know my client is HIV positive, the first thing that I will advocate for is his/her right to health. Once it is fulfilled by the police, then I will advocate for the rest. But right to health always comes first*.* (Paralegal 4)

This priority demonstrates the familiarity of LBHM’s paralegals with problems related to health. Having themselves experienced violation of rights impacting health on a daily basis, it is understandable that a main concern is the health status of their clients. This attention to the health condition of their clients, supports them to advocate for the right to health and other health-related rights.

### Raising awareness of right to health

Beyond checking clients’ health condition, paralegals also informed their clients about their right to health, and sometimes provided information related to health services available to their clients in closed settings.

The research found that twelve out of thirteen, or around 92% of the paralegals who participated in the study and had experience assisting clients who were detained in police custody gave their clients information about the right to health. Three of them always provided such information, and four of them provided such information frequently. A similarly high percentage of paralegals gave information on the right to health to clients held in a detention center or prison (87.5%). Table 3 provides the exact number of the paralegals who gave this kind of information to their clients.

**Table 3:** Paralegals give information about the clients’ right to health

Settings	Responses					Total
	Never	Seldom	Sometimes	Frequently	Always	
Police Detention	1 (8%)	3 (23%)	2 (15%)	4 (31%)	3 (23%)	13 (100%)
Detention Center	1 (12.5%)	2 (25%)	1 (12.5%)	2 (25%)	2 (25%)	8 (100%)
Correctional Facility	1 (12.5%)	2 (25%)	1 (12.5%)	2 (25%)	2 (25%)	8 (100%)

The provision of information related to the right to health and health services available at the detention center or correctional facility was usually the next step after the paralegal asked about their client’s health condition. In a situation where the client expressed concern about their health, paralegals would provide such information on the availability of health services. In a situation where the client did not have health issues, some paralegals reminded them that they too have the right to health. There were also situations where the clients themselves proactively asked about available health services.

Usually, my client asked me what health services are available in detention center or in correctional facilities. They asked, “What if I am sick?” I told them that in some detention centers or prisons, there is a civil society organization working together with the detention center. I told them, “You can ask to be put in touch with them, if for example, you are HIV positive and need treatment. They have the medicine.” (Paralegal 2)

### Ensuring health services in detention

Increasing access to health can also mean providing greater access to health facilities, goods, and services. Paralegals can take two approaches to ensure access to health services in detention centers or correctional facilities: taking the medicine directly to clients inside facilities or requesting the law enforcement agency to refer clients to health facilities. Table 4 presents the number of paralegals who went exta mile to ensure their client’s health-related rights.

**Table 4:** Paralegals ensure clients have access to medicine

Settings	Responses					Total
	Never	Seldom	Sometimes	Frequently	Always	
Police Detention	7 (53.5%)	0 (0%)	0 (0%)	1 (8%)	5 (38.5%)	13 (100%)
Detention Center	4 (50%)	0 (0%)	1 (12.5%)	0 (0%)	3 (37.5%)	8 (100%)
Correctional Facility	4 (50%)	0 (0%)	1 (12.5%)	1 (12.5%)	2 (25%)	8 (100%)

Six paralegals said that they ensured that their clients had access to medicines in police custody, and four paralegals did this both in the detention center and in the correctional facility. Five out of the six paralegals who ensured their clients had regular access to medicine in police custody did this on all of the cases s/he assisted, while another one only did it frequently.

Taking Medicine Directly To Clients

Out of thirteen paralegals who had experience in assisting a case in which the client was detained in police custody, six took medicine to their clients themselves. During assistance in detention centers and correctional facilities, 50% of the paralegals stated that they had delivered medicine to their clients.

One aspect that may affect paralegals’ ability to take medicine to their client is the paralegal’s main job. In some situations, LBHM’s paralegals worked as outreach workers or in a Community Health Clinic (CHC) where their clients access methadone or antiretroviral therapy. With their position, the paralegal was able to go the extra mile to help their clients without breaking any laws.

I usually help preparing some letters from the Community Health Care for my clients. For example, if my client is on methadone therapy, I will help make the methadone letter [that confirmed the client’s status as methadone patient]... I also help clients to enroll in the national health insurance system. (Paralegal 3)

Taking medicines to clients directly was not always easy. Paralegals’ position as outreach workers or in a CHC did not always guarantee that they could successfully take medicines to their clients. It required good negotiation skills with relevant law enforcement agencies. The more frequently paralegals advocated for taking medicines directly to the clients and the more exposed law enforcement agents became to information about detainees’ right to health, the more likely it was for such advocacy to be successful.

In the past few years, it is easier to advocate for the right to health (i.e. bringing medicine to a client who is detained). Maybe because the police have experienced similar requests before. But it was once very difficult to do so, especially before 2010. (Paralegal 4)

Encouraging Law Enforcement Agencies to Refer Clients to Health Services

Not all LBHM’s paralegals worked as outreach workers, or in a CHC. For those who did not, taking medicines directly to their clients was impossible as they did not have access to the medicines needed. To ensure access to health services in detention, some paralegals encouraged law enforcement agents to refer their clients to treatment, either for drug dependency or mental health treatment. However, the number of paralegals who did this was substantially lower than those who took medicines to their clients.

On average, only 30% of paralegals had ever asked law enforcement agents to refer their clients to drug dependency treatment, and only one paralegal had ever asked for his/her client to be referred to mental health facilities. According to paralegals’ testimony, the decision on whether or not to ask law enforcement agents to refer their clients to health facilities depended on the client’s need to be referred.

Not all clients need to be put in health faciliies. Some clients only needed paralegals’ help in ensuring that they had access to medication while in a closed setting. For a range of different reasons, some clients rejected the idea of paralegals asking law enforcement agents to refer them to health facilities. As paralegals’ actions must be based on the client’s consent, they could only encourage the clients to consider being referred to health facilities, but they could not request this if the client did not want to be referred.

One of my clients was infected with tuberculosis after he was detained. I asked Pak Tardus, the person who works at the detention’s health clinic, to put my client in Drug Dependency Hospital instead of the Kramat Jati Police Hospital. I told him that the facility in Kramat Jati Police Hospital is not good. Pak Tardus granted my request, and referred my client to Drug Dependency Hospital. (Paralegal 4)

Despite the low number of paralegals who advocated for their client to be referred to health facilities, the success rate was relatively high, ranging from 50 to 75%. Three paralegals claimed that they succeeded in getting police officers to refer their clients to health facilities; two paralegals claimed similar success with prosecutors; while one paralegal claimed s/he succeeded in encouraging prison officers to refer their clients to health facilities.

### Seeking an alternative to incarceration or lower sentence in legal defense

The right to health do not end when someone is found guilty of criminal charges. The stage when someone serves their sentence is usually longer than the pre-trial detention, and with the issue of overcapacity in prison, health risks are heightened. Here, paralegals play an important role to help clients seek an alternative to incarceration whenever possible, and permissible. Such help is given through intervention in the legal defense.

For many of LBHM’s paralegals working on drug-related cases, one of the objectives is to obtain a rehabilitation verdict for people who use drugs. The Narcotic Law allows judges to sentence people who use drugs to undergo rehabilitation services as an alternative to imprisonment.

Paralegal 4, a paralegal who specialized in drug cases said that he always tried to help obtain a rehabilitation verdict for his clients who had been proven to be involved only in drug use and not drug trafficking. He calculated that, during the many years of his paralegal work, he had helped many people to obtain rehabilitation rather than incarceration. He stated, “For rehabilitation, I can count around ten out of sixty cases that I handled so far [the client] got rehabilitation.”

One of the cases that stood out for him was a case involving Rose (not her real name), a woman who used drugs. Following her arrest for drug consumption, she kept using drugs while in detention. Due to this behavior, the lawyer who assisted Rose wanted to drop her case but the paralegal explained to the lawyer that this is precisely why Rose needed help: she had drug dependency and prison would not help her address her condition. In the court, he helped the lawyers explain Rose’s drug using behavior, and argued that Rose should have been given proper rehabilitation services. In the end, the judge sentenced her to rehabilitation facilities. Being asked about why the case was memorable for him, Paralegal 4 answered, “That case has a lesson. We find that if the convict does not receive her/his rights of rehabilitation, there will never be an assurance that s/he will not consume drugs again, due to her/his addiction.”

Another paralegal reported similar success when, in 2016, she assisted a drug user who regularly accessed methadone treatment in a local CHC. Her client was arrested for amphetamine possession. She pushed the local CHC to issue a letter about her client’s health situation as a patient there, and that he was still on treatment to cure his drug dependency when he was arrested. She then gave the letter to the judge. As a result, the client got a relatively lenient punishment of 1 year in prison, while the minimum sentence according to the Narcotic Law is 4years. Paralegal 3 reflected on her decision: “(If he was not assisted), he could get four years. That’s the minimum of drug user. That’s why his friends are confused. How could he get one year? It’s so short.” These experiences demonstrate how paralegals’ interventions in addressing access to justice significantly impact their clients’ health.

## Discussion

Trained and experienced paralegals have the potential to improve the respect, protection and fulfilment of clients’ health-related rights. The four staples of LBHM’s paralegals’ work reflect the tangible impacts paralegals bring into the life of marginalized community members. Their role in checking the clients’ health is similar to the role of paralegals as documenters of human rights abuses in detention [6]. Their activities in raising clients’ awareness on their own rights to health bring the same components of the ‘traditional’ role of legal empowerment [3, 5] but with focused training on the right to health. Similar to the achievements of paralegal programs in Sierra Leone, Rwanda, and Uganda [5, 17], paralegals in Indonesia succeeded in demanding pre-trial release or bail by highlighting the need for health treatment.

LBHM’s paralegals’ success in helping clients to obtain non-custodial sentences reflects previous findings where paralegals have been involved in projects aiming to obtain rehabilitation verdict for drug users. A project supported by the United Nations Office on Drugs and Crime in Indonesia sought to train paralegals from civil society organizations and legal aid organizations with the aim of improving their capacity to secure alternatives to incarceration such as rehabilitation [18]. Another recent paralegal program in Indonesia also recognized the gap in legal services for drug users and aims to provide adequate legal services through trained paralegals for a verdict of rehabilitation instead of prison time [19]. All of these efforts reflect the increasing understanding of the broad roles that paralegals can play to improve access to justice and health.

This research identifies several important factors for success. In respect to awareness raising, LBHM’s paralegals have the advantage of coming from the communities they serve, therefore, it is easier for the client to trust them. As fellow community members, paralegals have a greater sense of the problems faced by their clients, including health-associated risks and needs as the result of their case or of being detained. In some situations, LBHM’s paralegals even have the experience of having been detainees themselves. Previous bodies of literature show that by originating from marginalized communities, paralegals can work more effectively and attract more willingness from their community members to act [2, 10, 20, 21].

Another crucial factor for the success is their networks with healthcare providers and law enforcement agencies which allow them to go the extra mile in ensuring their clients’ health service needs. Some paralegals have regular jobs as health care workers and NGO staff which make them more knowledgeable about healthcare services and facilities. Their relationships with law enforcement agencies also enable them to negotiate clients’ bail or referral to health care facilities. This situation implies that in order to succeed, paralegals should build relationships with law enforcement and develop collaborative solutions [13]. Apart from networks with health care providers and law enforcement agencies, paralegals’ affiliation with LBHM is also beneficial for paralegals to obtain advice on how to deal with their cases; this affirms that partnership with local legal aid organizations is imperative for paralegal programs [12].

The need to incorporate paralegals into the global legal system where their work is acknowledged by the local context and civil law is well documented [2, 10, 22]. While the formal definition of paralegal has yet to be made clear, Indonesia has provided regulation on the role of paralegals through its Ministry of Law and Human Rights Regulation No. 1 Year 2018 on Paralegal in The Provision of Legal Aid. Although paralegals do not have direct obligations under the right to health, they can play a crucial role in supporting the health of their clients. This approach can in turn ensure government fulfill relevant obligations they have to promote and protect health. This is not to serve as a proxy for what governments ought to do, but instead to ensure governments fulfill their roles as a step towards repairing a broken system. In the future, the results of this research could provide a basis for governments to recognize and support the functions of paralegals, including in relation to enhancing the right to health.

The need for and potential significance of paralegals is increasing considering that globally, in 2016, there were at least 10.4 million people in prison as a result of criminal charges [23]. There are efforts focusing on increasing the standard of health among people in prison, such as the enactments of international standards which include the provision of adequate healthcare for prisoners under arrest or awaiting trial.^2^ However, countries where mass incarceration occurs and prisons are over-crowded, such as Indonesia, immense efforts are need to ensure that these standards move from paper to implementation. Based on the Ministry of Finance Regulation Number 64/PMK.02/2008, the government has cut the meal budget for prisoners to IDR 15,000 (approximately USD 1) per day for three meals. Many prisons, especially police detention centres, do not have in-house doctors. This situation is not unique to Indonesia and highlights the need for increased attention to health-related rights in these settings.

Alongside the above, there is a trend of using criminal charges as a ‘tool of social control to promote public safety, ‘morals’ and public health’ [24]. Such punitive approaches put socially excluded groups such as people who use drugs, lesbian, gay, bisexual, transgender and intersex persons, adolescents, women, and poor people, many of whom are already facing health challenges, at a higher risk of facing criminal charges. When this happens, these groups do not only need help in accessing justice, but also in accessing healthcare and facilities while facing charges. One of best practices in providing access to justice for socially excluded groups is through the use of community paralegals [25].

In such situations, some paralegals, mostly those who have access to health services or goods, play an important role in ensuring that clients can obtain the treatment they need. As not all paralegals interviewed in this research applied the same techniques to ensure their clients’ health, more training which includes practical exercises should be conducted. The training should also widen the scope of legal empowerment where paralegals can equip community members with relevant knowledge regarding health-related rights. Paralegals, and lawyers, might not always have access to the clients twenty-four hours a day. In many situations, clients have no choice other than negotiating their health needs directly with law enforcement agencies. When this happens, the client’s knowledge on the right to health becomes an effective tool for negotiation.

One of the general weaknesses of paralegal programs is the lack of management or coordination of paralegals once they have been trained. LBHM’s paralegal programs are not immune to this problem, shown by the inability to access many of the paralegals to participate in the research. Despite the positive changes paralegals can bring, many face problems in sustaining their efforts. The reasons for this include the limited possibilities to find stable sources of funding to make the program possible and to support their livelihood, particularly given the need to find the balance between sustainable paralegal interventions and the need for independence from donors and government [12].

It is beyond doubt that paralegals’ work benefits their clients’ legal rights. However, when LBHM started their paralegal initiatives, the benefit on the fulfilment of the health-related rights was not explicitly considered. Efforts to improve realization of the right to health of people in closed settings are often limited to the provision of doctors, facilities, or goods. This research strengthens the evidence base that paralegals can also become an additional avenue for enhancing detainees’ rights to health through checking clients’ health, raising awareness, ensuring health services in detention, and seeking for alternative or lower sentences where appropriate.

### Study limitations

This study has several limitations. This research is a self-assessment and did not include the perspectives of clients of the paralegals, therefore, the positive impacts reflected in this research might be exaggerated. Further, this research does not incorporate the perspective of health care workers, such as doctors and psychologists, to measure the medical effect of paralegals’ assistance on their clients’ health. Future research should attempt to combine perspectives from many parties to gain a more holistic picture of paralegals’ impact on the right to health.

## Conclusion

LBHM’s paralegal work has had significant positive impacts on the fulfilment of the health and other related rights of people who use drugs and other marginalized communities facing criminal processes in Jakarta, Indonesia. Particularly when working with marginalized populations, paralegals can go beyond simply facilitating access to justice to also help promote the health-related rights of their clients. Starting from LBHM’s model of paralegal engagement that includes also attention to health-related rights, relevant stakeholders from around the world might consider building a network of paralegals to address common issues, and regularly share their methods of work so that their skills in assisting clients can be regularly updated. Training on selected skills necessary in performing their duties as paralegals is also recommended. This training may include, but not limited to negotiation skills, writing up press releases/media pieces, and basic counseling.

## Data Availability

The datasets used and/or analyzed during the current study are available from the corresponding author on reasonable request.

## Abbreviations

CHC: Community Health Clinic
HIV: Human Immunodeficiency Virus
LGBTQI: Lesbian, Gay, Bisexual, Transgender, Queer, Intersex
LBHM: Lembaga Bantuan Hukum Masyarakat
PLHIV: People Living with HIV

## Footnotes

1 Calculated from those responding ‘Seldom’, ‘Sometimes’, ‘Frequently’ and ‘Always’.

2 These standards including, but not limited to, the revised Standard Minimum Rules for the Treatment of Prisoners and the UN Bangkok Rules.
